# Acute ethanol-induced liver injury is prevented by betaine administration

**DOI:** 10.3389/fphys.2022.940148

**Published:** 2022-10-04

**Authors:** Madan Kumar Arumugam, Srinivas Chava, Sathish Kumar Perumal, Matthew C. Paal, Karuna Rasineni, Murali Ganesan, Terrence M. Donohue, Natalia A. Osna, Kusum K. Kharbanda

**Affiliations:** ^1^ Research Service, Veterans Affairs Nebraska-Western Iowa Health Care System, Omaha, NE, United States; ^2^ Department of Internal Medicine, University of Nebraska Medical Center, Omaha, NE, United States; ^3^ Department of Biochemistry and Molecular Biology, University of Nebraska Medical Center, Omaha, NE, United States

**Keywords:** liver injury, ethanol binges, S-adenosylhomocysteine, S-adenosylmethionine, alcohol, betaine

## Abstract

Binge drinking is the most common form of excessive alcohol use. Repeated episodes of binge drinking cause multiple organ injuries, including liver damage. We previously demonstrated that chronic ethanol administration causes a decline in the intrahepatic ratio of S-adenosylmethionine (SAM) to S-adenosylhomocysteine (SAH). This decline causes impairments in essential methylation reactions that result in alcohol-induced fatty liver (steatosis) and other features of alcohol-associated liver disease (ALD). Co-treatment with betaine during chronic ethanol feeding, normalizes hepatocellular SAM:SAH ratio and alleviates many features of liver damage including steatosis. Here, we sought to examine whether betaine treatment similarly protects against liver injury in an alcohol binge-drinking model. We hypothesized that ethanol binge with prior or simultaneous betaine administration would prevent or attenuate acute alcohol-induced liver damage. Male C57Bl/6 mice were gavaged twice, 12 h apart, with either 6 g ethanol/kg BW or with an equal volume/kg BW of 0.9% NaCl. Two separate groups of mice (*n* = 5/group) were gavaged with 4 g betaine/kg BW, either 2 h before or simultaneously with the ethanol or saline gavages. All mice were sacrificed 8 h after the last gavage and serum and liver parameters were quantified. Ethanol binges caused a 50% decrease in hepatic SAM:SAH ratio and a >3-fold rise in liver triglycerides (*p* ≤ 0.05). These latter changes were accompanied by elevated serum AST and ALT activities and blood alcohol concentrations (BAC) that were ∼three-times higher than the legal limit of intoxication in humans. Mice that were treated with betaine 2 h before or simultaneously with the ethanol binges exhibited similar BAC as in mice given ethanol-alone. Both betaine treatments significantly elevated hepatic SAM levels thereby normalizing the SAM:SAH ratio and attenuating hepatic steatosis and other injury parameters, compared with mice given ethanol alone. Simultaneous betaine co-administration with ethanol was more effective in preventing or attenuating liver injury than betaine given before ethanol gavage. Our findings confirm the potential therapeutic value of betaine administration in preventing liver injury after binge drinking in an animal model.

## Introduction

Heavy drinking is an important social, economic, and clinical problem (Axley et al., 2019). In humans, alcohol intake can be acute (single occasion over several hours), short-term (for several days), or long-term/chronic (for years/decades). Binge drinking is the most common, costly, and deadly pattern of excessive alcohol use in the United States (Esser et al., 2014; Stahre et al., 2014; Sacks et al., 2015). The National Institute on Alcohol Abuse and Alcoholism defines binge drinking as a pattern of drinking that brings a person’s blood alcohol concentration (BAC) to 0.08 g per 100 ml or greater after five or more drinks (12.5 g ethyl alcohol per drink) in about 2 h (Ventura-Cots et al., 2017). There are several detrimental consequences of binge drinking that affect the individual and society. There are immediate risks, including personal injury, driving accidents, unwanted pregnancies, and death due to alcohol overdose. Longer-term risks include repeated episodes of binge drinking that cause not only detrimental neurobiological consequences but also generate adverse effects on almost all organ systems including heart, liver, immune system, bone health and gastrointestinal tract (Crabbe et al., 2011).

The liver is the principal organ of alcohol metabolism and a major target organ of acute and chronic alcohol-induced injury (Osna et al., 2017). The susceptibility of the liver to alcohol-induced toxicity is due to both the high concentrations of alcohol found in the portal blood (versus systemic) as well as the metabolic consequences of ethanol metabolism (Osna et al., 2017). Our current understanding of the effects of binge drinking on liver injury is not as complete as that regarding the effects of chronic ethanol exposure. However, there are, some parallels between acute and chronic alcohol exposure (Massey and Arteel, 2012). Acute alcohol exposure strategies are classified as single bolus dose models, multiple bolus dose models, and “2-hit” models. All these develop degrees of liver injury similar to those in animals subjected to chronic ethanol administration.

Our laboratory has reported that ethanol consumption causes liver injury by altering methionine metabolism specifically by causing a decline in methionine synthase (MS), a critical enzyme that remethylates homocysteine. (Kharbanda and Barak, 2005; Kharbanda, 2009, 2013). We later reported that the most detrimental effect of the ethanol-induced decrease in MS is the decline in hepatic S-adenosylmethionine (SAM) and a concurrent rise in S-adenosylhomocysteine (SAH) (Kharbanda and Barak, 2005; Kharbanda et al., 2007a; Kharbanda, 2009, 2013). The decline in hepatic SAM:SAH ratio impairs several essential methylation reactions, leading to the development of liver injury (Kharbanda and Barak, 2005; Kharbanda et al., 2005; Kharbanda et al., 2007a; Kharbanda et al., 2007b; Kharbanda, 2007; Kharbanda, 2009, 2013; Ganesan et al., 2016). Additional studies demonstrated that betaine is a crucial methyl group donor and key regulator of the methionine cycle and when co-administered with alcohol, ameliorates a number of hallmark features of liver injury (Ji and Kaplowitz, 2003; Ji et al., 2004; Kharbanda et al., 2005; Kharbanda et al., 2007a; Kharbanda et al., 2007b). We further showed that such alleviation of alcohol-induced liver injury occurs because betaine treatment normalizes the hepatocellular SAM: SAH ratio to restore cellular methylation reactions (Ji and Kaplowitz, 2003; Ji et al., 2004; Kharbanda et al., 2005; Kharbanda et al., 2007a; Kharbanda et al., 2007b) by enhancing betaine homocysteine methyltransferase (BHMT) expression and activity (Kharbanda et al., 2007a). This enzyme utilizes betaine to catalyze an alternate pathway reaction that remethylates homocysteine, thereby removing SAH, regenerating SAM and maintaining SAM:SAH homeostasis (Kharbanda and Barak, 2005; Kharbanda, 2009, 2013). Here, our aim was to ascertain whether betaine administration attenuates or prevents acute alcohol-induced liver damage in mice and, if effective, determine betaine’s mechanism of action. Based on our observations that betaine prevents liver injury after chronic ethanol exposure (Ji and Kaplowitz, 2003; Ji et al., 2004; Kharbanda et al., 2005; Kharbanda et al., 2007a; Kharbanda et al., 2007b), we hypothesized that betaine treatment, given before or at the time of an alcohol binge, would similarly prevent the development of alcohol-induced liver injury.

## Materials and methods

### Ethanol-binge model

Ten-week-old male C57Bl/6J mice were purchased from Jackson laboratories (Bar Harbor, ME, United States). The mice were housed in our Animal Research Facility for 1 week and then gavaged twice, 12 h apart (beginning at 7 p.m. and at 7 a.m. the following day), with either 0.9% NaCl or with 6 g ethanol/kg BW, following the protocol of Leung *et al.* (Leung et al., 2012). Two other groups of mice (*n* = 5 each) were gavaged with betaine (4 g betaine/kg BW), either 2 h before (i.e., beginning, at 5 p.m. and at 5 a.m. the following day) the ethanol gavages or simultaneously with ethanol, the latter using an ethanol-betaine mixture. All mice were sacrificed 8 h after the second saline/ethanol gavage. The care, use, and procedures performed on these mice were approved by the Institutional Animal Care and Use Committee at the Omaha Veterans Affairs Medical Center.

During animal sacrifice, blood was collected from each mouse and the liver was removed. Serum was prepared by centrifuging whole blood in serum separator tubes at 13,000 X g for 5 min. Portions of each liver were immediately fixed in formalin for histology or processed for the preparation of a deproteinized extract using perchloric acid for HPLC analysis of SAM and SAH, as detailed (Kharbanda et al., 2007a). The remainder of each liver was freeze-clamped and stored at −70°C for subsequent biochemical assays.

### Serum ethanol, aspartate transaminase and alanine transaminase levels

We quantified serum ethanol concentrations by gas chromatography, using an Agilent GC 3800 system (Donohue et al., 2007). Serum AST and ALT activities, standard markers of liver injury, were measured by the clinical chemistry laboratory at the Omaha Veterans Affairs Medical Center, using the VITROS 5.1 FS Chemistry System (Ortho Clinical Diagnostics, Raritan, NJ).

### Hepatic histology

Formalin-fixed liver sections were prepared, stained with hematoxylin and eosin and assessed for pathological changes. Digital images were acquired using a Keyence BZ-X810 microscope (Plano, TX, United States).

### Hepatic SAM, SAH and triglyceride levels

We subjected liver perchloric acid extracts to high-performance liquid chromatography (HPLC) to quantify SAM and SAH levels, as detailed (Kharbanda et al., 2007a; Kharbanda et al., 2014).

We quantified triglycerides in liver lipid extracts (Folch et al., 1957) using the diagnostics kit (Cat#TR22421, Thermo Electron Clinical Chemistry, Louisville, CO, United States) using the manufacturer’s instructions, as detailed previously (Kharbanda et al., 2007a).

### Reactive oxygen species and thiobarbituric acid-reactive substances

Liver ROS were measured by using 2′7′-dichlorodihydrofluorescein diacetate (DCFH-DA) as detailed (Rodrigues Siqueira et al., 2005). Formation of the oxidized fluorescent derivative, dichlorofluorescein (DCF), was monitored at of 485 nm (excitation) and 530 nm (emission). Data are expressed as fluorescence units and are normalized for protein concentration, measured by the Bradford dye-binding assay (Bradford, 1976).

We measured hepatic lipid peroxidation by TBARS assay, as detailed (Uchiyama and Mihara, 1978) using purified malondialdehyde (MDA) as the standard. Data are expressed as pmoles TBARs equivalents and were normalized for protein concentration (Bradford, 1976).

### Proteasome activity

Liver homogenates were used to assay the chymotrypsin-like activity of the proteasome (Suc-LLVY-AMC hydrolysis) as previously described (Osna et al., 2008; Osna et al., 2010). Enzyme activities are expressed as nanomoles of 4-amino, 7-methyl coumarin formed per hour and specific enzyme activity was calculated after normalizing for protein concentration and expressed as nanomoles per mg protein (Bradford, 1976).

### Messenger RNA quantification

Total RNA was isolated from liver tissue (100 mg, RNA later treated) using PureLink™ RNA mini kit (Cat#12183018A, Invitrogen, Waltham, MA, United States), following the manufacturer’s instructions. RNA was quantified spectrophotometrically (NanoDrop Technologies, Wilmington, DE, United States) and 200 ng of RNA were reverse transcribed to cDNA using the high-capacity reverse transcription kit (Cat#4368813, Applied Biosystems, Waltham, MA, United States). We quantified the relative levels of the mRNAs encoding MS and BHMT, using TaqMan Universal Master Mix-II (Cat#4440038, Applied Biosystems, Waltham, MA, United States) with fluorescent-labeled FAM primers (TaqMan gene expression systems, Cat#4331182, Applied Biosystems, Waltham, MA, United States), using Model 7,500 Real-Time PCR Detection System (Applied Biosystems, Waltham, MA, United States). The relative quantity of each RNA transcript was calculated by its threshold cycle (Ct) after subtracting that of the reference cDNA (β-actin). Data are expressed as the relative quantity (RQ) of each transcript.

### Preparation of subcellular fractions

Liver pieces were homogenized in cold 5 mmol/L Tris (pH 7.4) containing 0.25 mol/L sucrose and 1 mmol/L EDTA and subcellular fractions prepared as detailed in our previous publication (Kharbanda et al., 2007b). Briefly, the homogenates were centrifuged at a low-speed spin (570 X g for 10 min) at 4°C. The supernatants obtained were centrifuged at 8500 X g for 20 min at 4°C to pellet the mitochondrial/lysosomal fractions. The resulting supernatants were then centrifuged at 105,000 X g for 60 min at 4°C to pellet the microsomes and yield the cytosol fractions (supernatants). All fractions were resuspended in an appropriate volume of the buffer.

### Ethanol metabolism

Alcohol dehydrogenase (ADH) a cytosol enzyme and cytochrome P450 2E1 (CYP2E1), a microsomal enzyme are the predominant enzymes that catalyze hepatic ethanol oxidation. We measured the catalytic activities of ADH and CYP2E1 in liver cytosol and microsomes fractions, respectively as detailed (Wu and Cederbaum, 1996; Chen and Cederbaum, 1998; Yan et al., 2006).

### Lysosomal hydrolases

Two major hepatic lysosomal hydrolases are cathepsin L, a serine proteinase, and the lysosomal acid lipase (LAL), which degrades triglycerides and cholesteryl esters (Barrett and Kirschke, 1981; Yan et al., 2006). We determined the catalytic activities of these two hydrolases in the homogenates and lysosomal fractions.

### Western blotting

We subjected liver homogenates to Western blot analysis, using primary antibodies directed against BHMT, glycine N-methyltransferase (GNMT) or *β*-actin, as we previously described (Kharbanda and Barak, 2005; Kharbanda et al., 2007a). We visualized the proteins using enhanced chemiluminescence detection. The intensities of immunoreactive protein bands were quantified using Quantity One software (Bio-Rad Laboratories, Hercules, CA).

### Statistical analysis

Data were analyzed by ANOVA followed by Tukey post-hoc test for comparisons among groups and results considered statistically different at a probability (*p*) value ≤0.05.

## Results

Acute ethanol treatment with or without either betaine treatment had no effect on the body or liver weight compared with saline-gavaged mice (Table 1).

**TABLE 1:** Effect of ethanol binges with or without betaine treatment on body and liver weight of control and experimental group of mice gavaged with saline (C), ethanol (E), or given betaine 2 h before (EB-1) or simultaneously (EB-2). With the ethanol binge. Data is presented as the mean ± SD (*n* = 5); data not sharing a common letter (superscript) significantly differ from each other at *p* ≤ 0.05.

	C	E	EB-1	EB-2
Body Weight (g)	22.26 ± 1.01a	21.90 ± 2.11a	22.12 ± 1.09a	22.00 ± 1.44a
Liver Weight (g)	1.09 ± 0.06a	1.05 ± 0.16a	1.13 ± 0.72a	1.06 ± 0.18a

### Blood alcohol, AST and ALT levels

**FIGURE 1:**
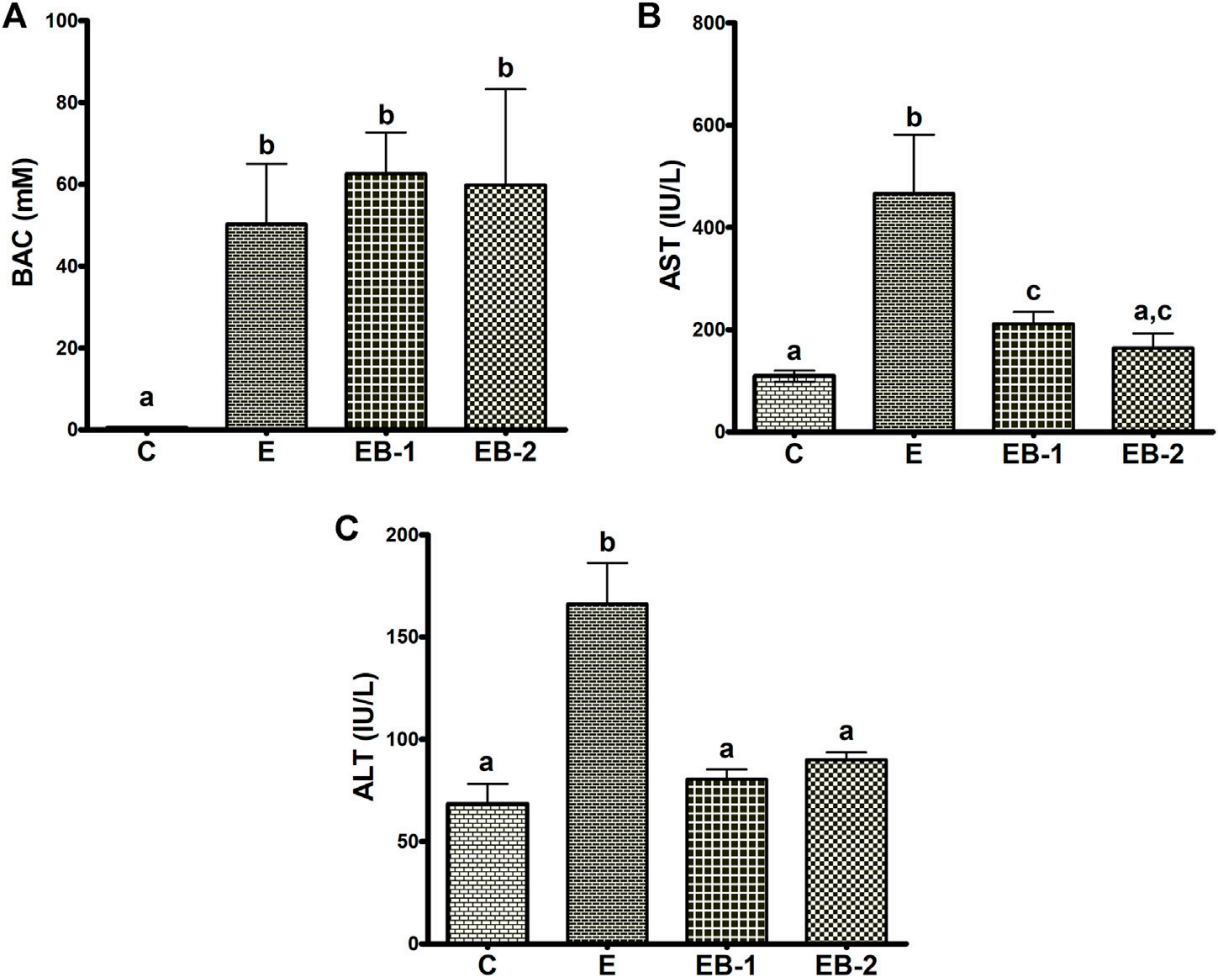
Blood alcohol, AST and ALT levels. **(A)** Serum blood alcohol concentration (BAC), **(B)** AST and **(C)** ALT levels in the livers of mice gavaged with saline [C], ethanol [E], or given betaine 2 h before [EB-1] or simultaneously with the ethanol binge [EB-2]. Data are presented as the mean ± SEM (*n* = 5); values not sharing a common letter significantly differ from each other at *p* ≤ 0.05.

Ethanol binges elevated the terminal BAC 3- to 4-fold higher than the legal limit (in humans) of 17.4 mM (80 mg/100 ml). The BAC in mice given betaine 2 h prior (EB-1) or at the same time as the ethanol binges (EB-2) showed the same ethanol levels as those given ethanol alone (Figure 1A). We determined the effects of acute alcohol binges, with or without betaine, on liver injury by assessing AST and ALT, the two serum biomarkers of liver injury. Compared with controls, serum ALT and AST activities were 2-3-fold higher in mice that received ethanol binges (Figures 1B,C). Betaine administered 2 h before (EB-1) or at the same time as the ethanol binges (EB-2) caused a significant reduction in serum AST and ALT, indicating attenuated liver injury (Figures 1B,C). Betaine given simultaneously with ethanol almost completely prevented the ethanol-induced rise in these enzymes, which remained essentially equal to control levels (Figures 1B,C). However, while AST levels were significantly lower in mice given betaine 2 h before the ethanol binge, compared with ethanol-treated mice, they were still significantly higher than serum AST levels in control mice (Figure 1B).

### Liver histology (H&E)

**FIGURE 2:**
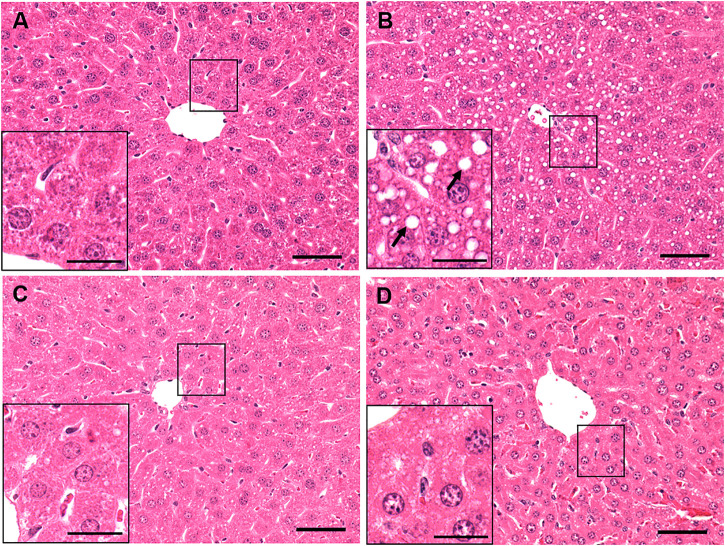
Histological assessment of liver pathology by hematoxylin and eosin (H and E) staining of a representative liver section of mice gavaged with **(A)** saline, **(B)** ethanol **(C)**, given betaine 2 h before ethanol (EB-1) or **(D)** simultaneously with the ethanol binge (EB-2). Macrovesicular lipid droplets (arrows) illustrating fat accumulation is seen only in the liver section of ethanol-fed representative mouse. Scale bar = 50 µm (magnified image scale bar = 20 µm).

Hematoxylin and eosin-stained liver sections of control mice (Figure 2A) showed normal histology, with no enlarged lipid droplets, whereas those of ethanol-treated mice revealed vacuolated areas predominantly in Zone 3, indicating fat accumulation in hepatocytes around the central vein (Figure 2B). However, there was no evidence of hepatic steatosis in either group of betaine-treated mice, as their liver histology was similar to that of saline-gavaged control mice (Figures 2C,D).

**FIGURE 3:**
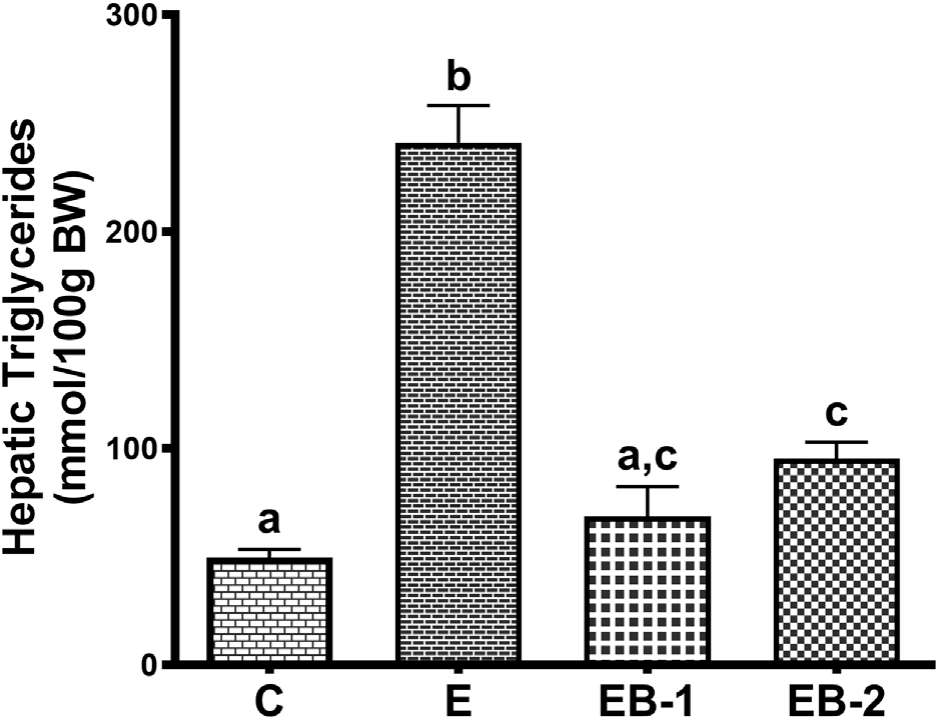
Hepatic triglyceride levels in mice gavaged with saline (C), ethanol (E), or given betaine 2 h before (EB-1) or simultaneously with the ethanol binges (EB-2). Data are presented as the mean ± SEM (*n* = 5); values not sharing a common letter significantly differ from each other at *p* ≤ 0.05.

Quantification of liver triglyceride levels corroborated our histological results. The elevated triglyceride levels seen in livers of ethanol-fed rats were significantly lower in both ethanol-betaine-treated groups (Figure 3).

### Hepatic SAM, SAH and SAM:SAH ratio

**FIGURE 4:**
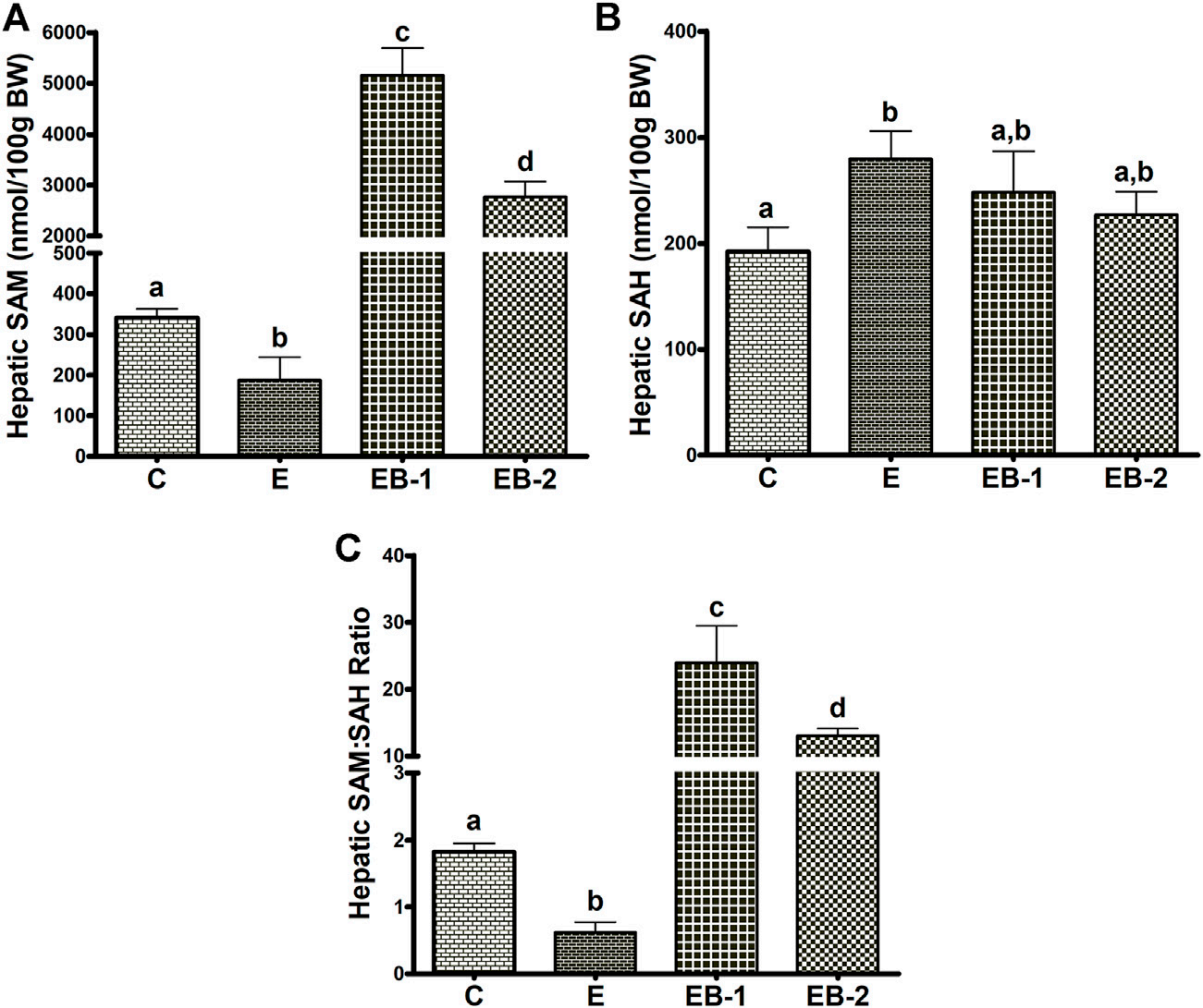
Hepatocellular SAM, SAH, and SAM:SAH ratio. **(A)** SAM, **(B)** SAH and **(C)** the calculated SAM:SAH ratio in livers of mice gavaged with saline [C], ethanol [E], or given betaine 2 h before [EB-1] or simultaneously with the ethanol binges [EB-2]. Data are presented as the mean ± SEM (*n* = 5); values not sharing a common letter significantly differ from each other at *p* ≤ 0.05.

Ethanol-gavaged mice showed significantly lower levels of hepatic SAM than saline-gavaged control mice (Figure 4A). Mice gavaged with betaine 2 h before (EB-1) or simultaneously with ethanol (EB-2) had significantly higher SAM levels than both control mice or mice given ethanol alone (Figure 4A). We observed a larger increase in SAM levels in mice given betaine 2 h prior to the ethanol binges.

Acute ethanol-treated mice exhibited a modest but significant rise in hepatic SAH levels, compared with saline-gavaged control mice (Figure 4B). Betaine given 2 h before (EB-1) or simultaneously with the ethanol binges (EB-2) caused a numeric but insignificant decrease in hepatic SAH compared to ethanol-treated mice (Figure 4B). This decline resulted in SAH levels in both betaine-treated groups that were also comparable to those of saline-treated control mice.

Because of the modest changes in hepatic SAH levels across the groups, the calculated ratio of SAM:SAH followed a similar pattern as the SAM levels. We observed a lower SAM:SAH ratio in ethanol gavaged mice but a 5- to 10-fold higher SAM:SAH ratio in both betaine treated groups compared with controls (Figure 4C).

### BHMT and MS expression

**FIGURE 5:**
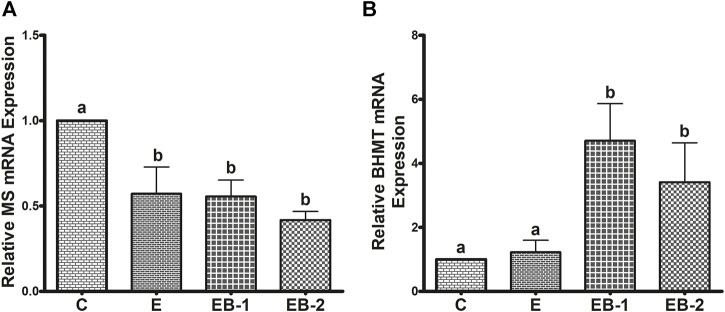
**(A)** MS and **(B)** BHMT mRNA quantification by qPCR. Levels of MS and BHMT mRNA in mice treated with saline [C], ethanol [E], or given betaine 2 h before [EB-1] or simultaneously with the ethanol binges [EB-2]. Data are presented as mean values ± SEM (*n* = 5); values not sharing a common letter significantly differ from each other at *p* ≤ 0.05.

To understand the reason for the decrease in SAM levels by ethanol binge and its very robust restoration after betaine treatment, we quantified two enzymes, BHMT and MS that catalyze separate reactions to remethylate homocysteine for removing SAH, regenerating SAM, and maintaining a relatively constant SAM:SAH ratio in the liver (Kharbanda and Barak, 2005; Kharbanda, 2009, 2013). Ethanol binges reduced liver MS mRNA content by nearly 50% (Figure 5A) but had no effect on BHMT mRNA, compared with that in control mice (Figure 5B). Neither betaine treatment affected the ethanol-elicited reduction in MS mRNA, as this remained nearly equal to that in the ethanol-treated group (Figure 5A). However, both betaine treatments significantly increased BHMT mRNA compared with controls or ethanol-binged mice (Figure 5B).

**FIGURE 6:**
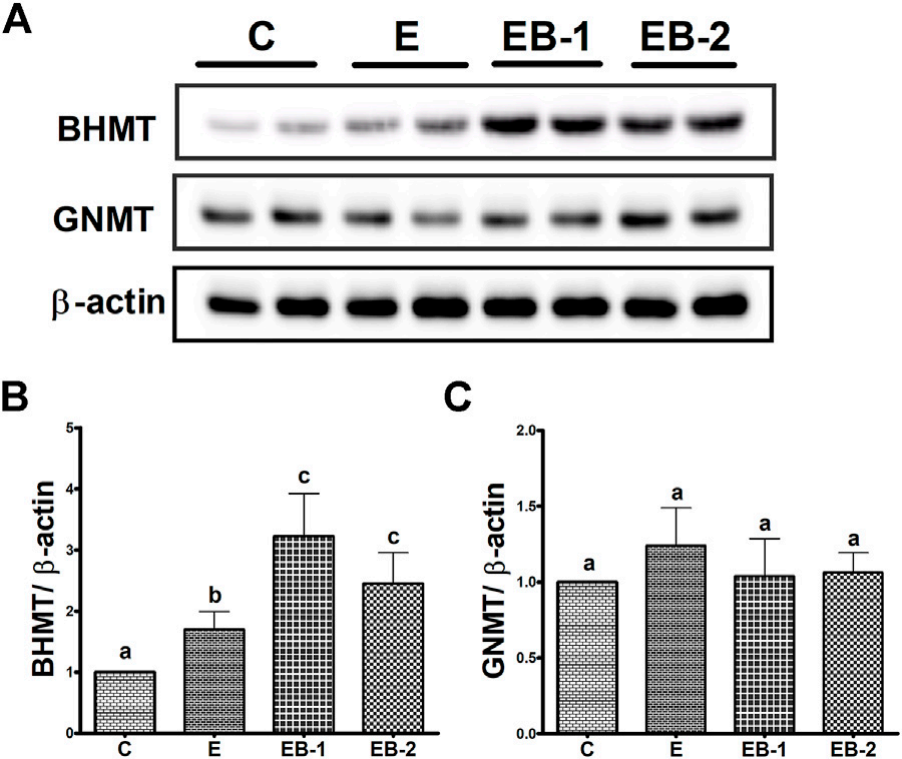
BHMT and GNMT protein quantification by Western blot. Liver homogenates of mice gavaged with saline [C], ethanol [E], or given betaine 2 h before [EB-1] or simultaneously with the ethanol binges [EB-2] were subjected to Western blot analysis. **(A)** Western Blot showing BHMT and GNMT expression in representative samples. **(B)** Immunoblot analyses summarizing the protein band density of BHMT to *β*-actin. **(C)** Immunoblot analyses summarizing the protein band density of GNMT to *β*-actin. Data are presented as mean values ± SEM (*n* = 5); values not sharing a common letter significantly differ from each other at *p* ≤ 0.05.

In contrast to comparable liver BHMT mRNA levels in control and ethanol-binged mice (Figure 5B), an increase in its protein levels (Figures 6A,B) was seen in the ethanol binged mice compared with controls. This rise was further elevated by both EB-1 and EB-2 treatments (Figures 6A,B). However, BHMT mRNA and its protein expression in EB-1 and EB-2 were not significantly different from each other (Figures 5B, 6A,B). Neither ethanol nor betaine treatments significantly affected GNMT protein levels over those of controls (Figure 6C).

### ROS, TBARS and proteasome activity

**FIGURE 7:**
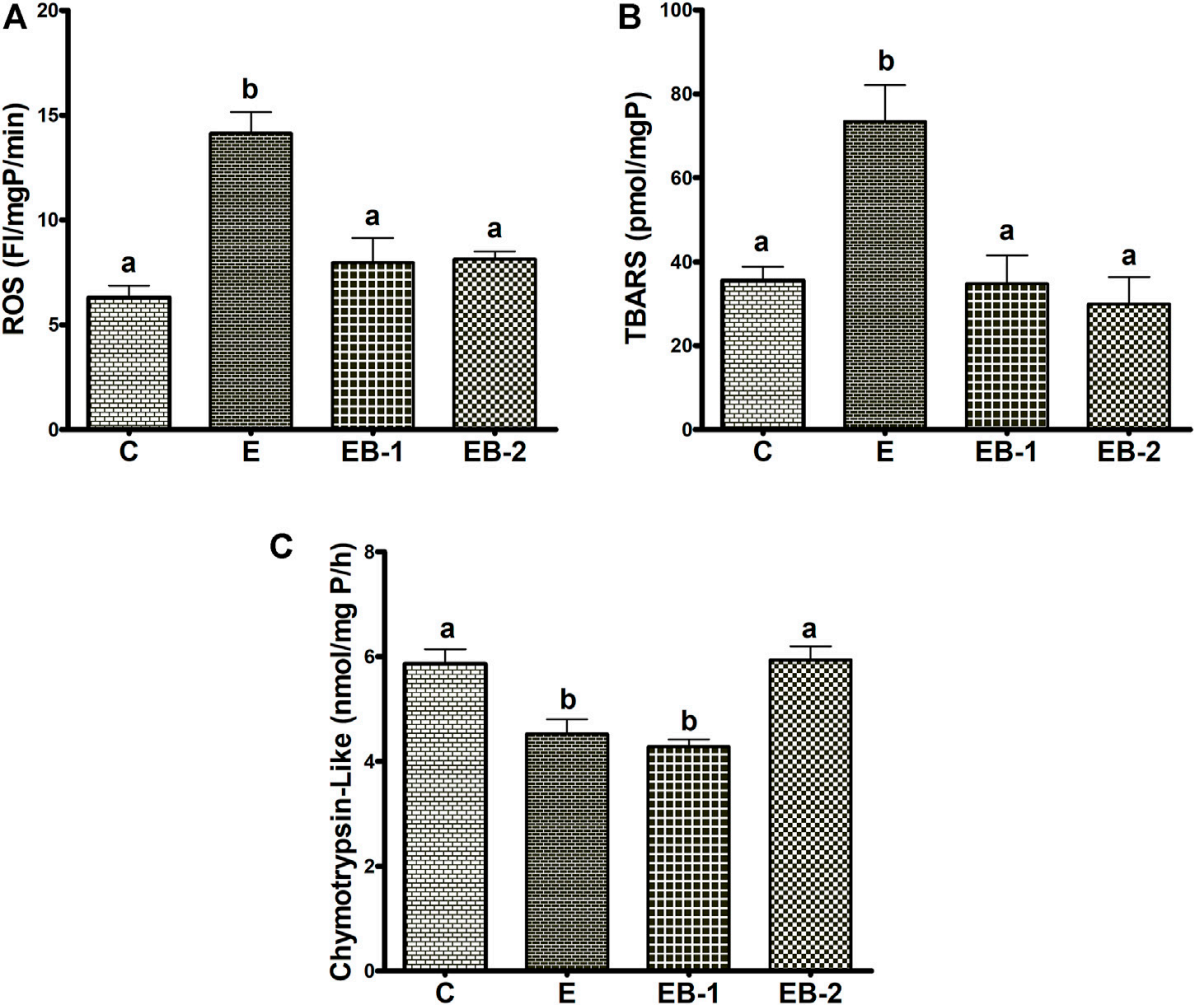
ROS, TBARS and proteasome chymotrypsin-like activity. Liver homogenates of mice gavaged with saline [C], ethanol [E], given betaine 2 h before [EB-1] or simultaneously with ethanol binges [EB-2] were quantified for **(A)** ROS, **(B)** TBARS and **(C)** Chymotrypsin-like proteasome activity. Data are presented as mean values ± SEM (*n* = 5); values not sharing a common letter significantly differ from each other at *p* ≤ 0.05.

We observed significant elevations in both ROS and TBARS after ethanol binges (Figures 7A,B). In both betaine-treated groups, ROS and TBARS remained equal to those in control (saline-treated) animals (Figures 7A,B). Hepatic chymotrypsin-like proteasome activity in livers of ethanol-treated mice declined by 25% compared with controls. Betaine administered 2 h before the ethanol binges (EB-1) did not restore proteasome activity to control levels, however proteasome activity was fully restored to normal in mice given betaine simultaneously with ethanol (EB-2; Figure 7C). Proteasome trypsin-like activity among all treatment groups was comparable (data not shown).

### Enzymes of ethanol oxidation

**FIGURE 8:**
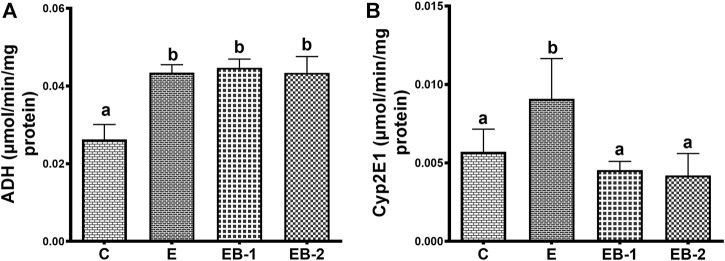
**(A)** Cytosolic ADH and **(B)** microsomal Cyp2E1 activity in the liver of mice gavaged with saline [C], ethanol [E], given betaine 2 h before [EB-1] or simultaneously with ethanol binges [EB-2]. Data are presented as mean values ± SEM (*n* = 5); values not sharing a common letter significantly differ from each other at *p* ≤ 0.05.

Acute ethanol administration to mice increased the specific activity of liver ADH 1.7-fold over that in saline-treated mice. Betaine administered either administered 2 h prior (EB-1) or simultaneously with ethanol (EB-2) did not reverse the rise in hepatic ADH (Figure 8A). Hepatic CYP2E1 specific activity in ethanol-gavaged mice was 1.5-fold higher than in saline-treated control animals. Both methods of betaine administration (EB1 and EB2) prevented the ethanol-induced rise in liver CYP2E1 (Figure 8B).

### Activities of lysosomal hydrolases

**FIGURE 9:**
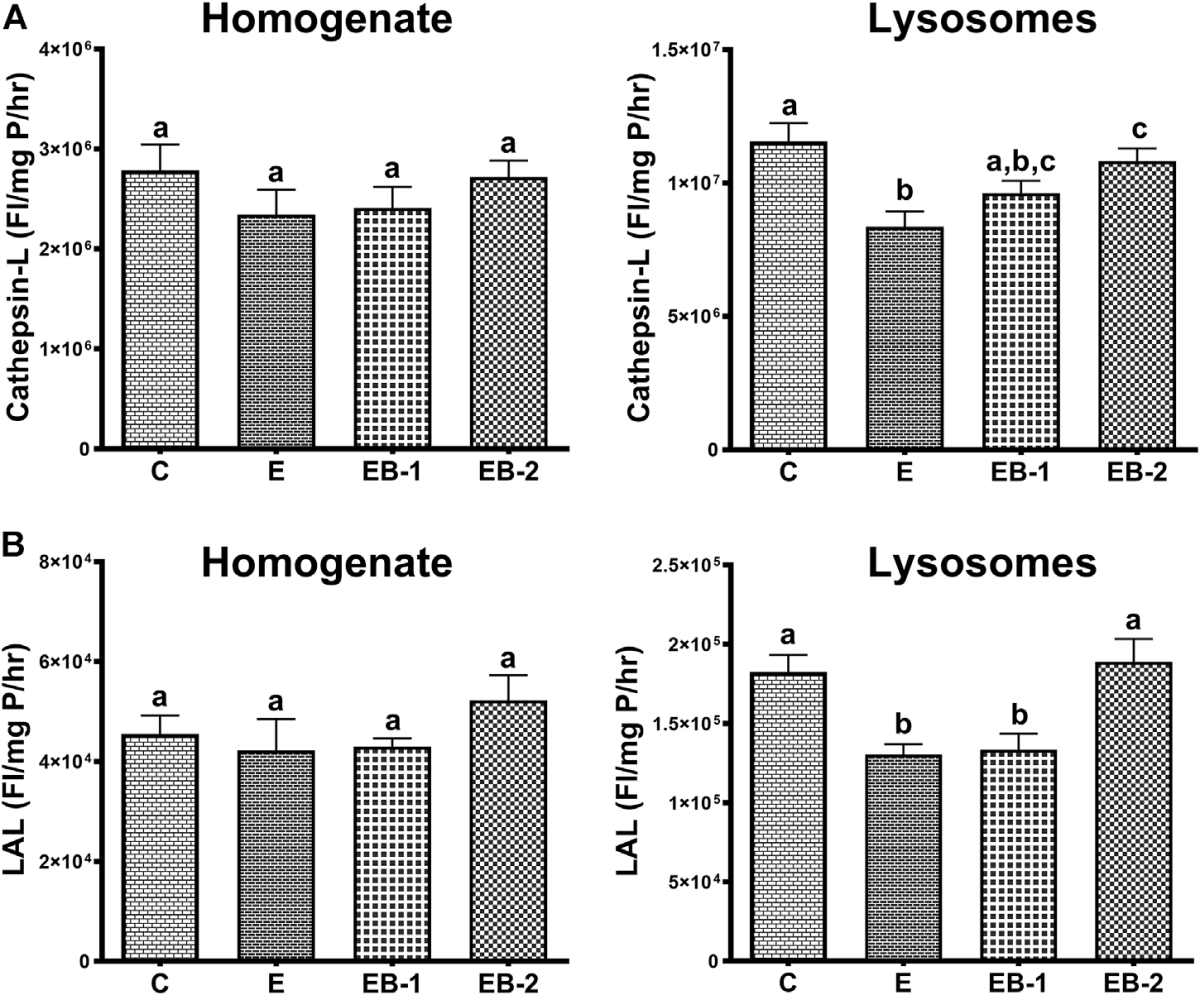
**(A)** Cathepsin-L and **(B)** LAL activities in the homogenate and lysosomal liver fractions of mice gavaged with saline [C], ethanol [E], given betaine 2 h before [EB-1] or simultaneously with ethanol binges [EB-2]. Data are presented as mean values ± SEM (*n* = 5); values not sharing a common letter significantly differ from each other at *p* ≤ 0.05.

Compared with saline-treated control mice, acute ethanol administration caused a 1.5-fold decrease in cathepsin L activity (Figure 9A) and a 1.3-fold loss of LAL activity (Figure 9B) in the lysosomal liver fractions. Betaine treatment either 2 h prior (EB-1) or simultaneously with ethanol gavage (EB-2) restored their activities to control levels. However, it is noteworthy that the degree of restoration of both lysosomal enzyme activities was greater when betaine was simultaneously administered with ethanol compared to 2 h prior (Figures 9A,B).

## Discussion

Alcohol exerts its toxic effects through multiple mechanisms including acetaldehyde toxicity, oxidant stress, endotoxins, cytokines, chemokines, a compromised immune system, and nutritional deficiencies (Lieber, 1995). Studies from our laboratory and those of others have shown that ethanol-induced disruption of methionine metabolism plays a pathogenic role in the development of ALD, primarily by lowering the hepatocellular SAM:SAH ratio (Halsted et al., 2002; Lu et al., 2002; Kharbanda et al., 2007a; Kharbanda, 2007; Kharbanda, 2009, 2013). The amount of alcohol consumed, and the duration of its consumption are the two significant factors that affect the degree of hepatic dysfunction. In addition, the means by which alcohol is delivered is also important in determining its pathological consequences (Brandon-Warner et al., 2012). Here, we used two ethanol binges 12 h apart and sacrificed the mice 8 h after the last gavage. This treatment regimen resulted in BAC levels that exceeded the legal limit in humans by more than three-fold and which generated the anticipated liver toxicity, as evidenced by elevated serum AST and ALT levels, which were accompanied by higher levels of hepatic oxidant stress, as judged by elevated ROS and TBARS (Figure 7). Morphological examination of hematoxylin- and eosin-stained liver sections from ethanol-binged mice revealed lipid droplets that were greater in numbers and larger in size than in controls. These histology findings were corroborated biochemically, showing that hepatic triglyceride levels in ethanol-gavaged mice were five-times higher than controls. However, 2 h prior treatment or co-treatment with betaine, a vital methylating agent (Craig, 2004; Kharbanda, 2009; Lever and Slow, 2010; Kharbanda, 2013), protected the liver from the toxic effects of ethanol by significantly attenuating the ethanol-induced rise in serum AST and ALT, while simultaneous or prior betaine treatment did not alter their BAC. Protection by betaine was also evident after pathological evaluation of liver damage as well as the nearly complete prevention of steatosis. These effects of betaine treatment are consistent with earlier findings in our and other laboratories using rodent models subjected to chronic ethanol feeding (Ji and Kaplowitz, 2003; Ji et al., 2004; Kharbanda et al., 2007a; Ji et al., 2007; Kharbanda, 2007; Ji et al., 2008; Kharbanda et al., 2009a; Kharbanda et al., 2012).

To clarify the mechanism(s) responsible for protection by betaine from ethanol-induced liver injury, we investigated whether acute ethanol administration alters either or both the metabolites and enzymes of the methionine metabolic pathway, as reported after chronic ethanol exposure (Ji et al., 2004; Kharbanda et al., 2007a). Here, acute ethanol administration to mice significantly decreased hepatic SAM levels by 50%, compared with control mice. The latter decrease likely resulted from the alcohol-induced reduction in MS expression. A similar reduction in MS expression was reported after 4 wk of chronic feeding to mice and rats (Ji et al., 2004; Kharbanda et al., 2007a). However, both betaine treatments used here, prevented the alcohol-induced decrease in SAM, which robustly exceeded control levels. These findings are consistent with previous reports from our and other laboratories, demonstrating increased hepatic SAM levels in animals fed a betaine-supplemented ethanol diet (Ji et al., 2004; Kharbanda et al., 2007a). We further demonstrated that the rise in hepatic SAM levels after betaine treatments were likely caused by increased BHMT mRNA and protein expressions, resulting in higher BHMT content and activity (Kharbanda et al., 2007a). Indeed, here we also observed, significant rises in BHMT mRNA and protein after both types of betaine treatments. However, while we reported a 4-fold rise in SAM levels over control rats chronically fed a betaine-supplemented ethanol diet (Kharbanda et al., 2007a), here, we observed a 14- or 8-fold rise in SAM over controls when betaine was administered to mice 2 h before or simultaneously with ethanol binges, respectively. This difference in the magnitude of increase in SAM levels by betaine in our previous chronic rat study vs*.* the present acute study here, is likely related to the different animal models, as comparable rises in SAM seen here were reported earlier in mice chronically fed a betaine-supplemented ethanol diet (Ji et al., 2004).

Here, SAH levels increased ∼1.5-fold in livers of ethanol-gavaged compared with saline-treated mice. This change was similar to that reported using the chronic ethanol feeding rodent models (Ji et al., 2004; Kharbanda et al., 2007a). Further, while both betaine treatments numerically lowered the ethanol-induced rises in SAH levels, they were not significantly different from either control or ethanol-treated mice. As a result of the moderate changes in SAH among the 4 groups, the calculated hepatic SAM:SAH ratio followed the same pattern as that of hepatic SAM levels, with greater than 10- and 5-fold increases over controls with betaine administered 2 h prior to or simultaneously with ethanol, respectively. We did not anticipate these rather dramatic rises in SAM:SAH ratios in acutely-betaine treated mice, as we observed comparable hepatic SAM:SAH ratios in rats fed a control or betaine-supplemented control/ethanol diet in our previously-reported chronic study (Kharbanda et al., 2007a). We believe that this dramatic rise in SAM levels and the mean SAM:SAH ratios over those of controls in betaine-treated mice is likely related to similar GNMT levels across all groups seen here. GNMT is regarded as a “sink” for “excess” intrahepatic SAM. The increase in GNMT by betaine treatments in our previous chronic ethanol study with rats (Kharbanda et al., 2009b) was likely responsible for enhancing the utilization of excess SAM to maintain hepatocellular SAM:SAH ratio as seen in control rats (Kharbanda et al., 2007a). It is noteworthy that the high SAM:SAH ratio after betaine co-treatment, as seen here, was also reported in a previous chronic ethanol mouse study (Ji et al., 2004).

In the present study, we also observed that acute ethanol gavage increased both ROS and TBARS while it lowered proteasome chymotrypsin-like-activity. Because the liver is the main site of ethanol metabolism, the ethanol-induced decrease in hepatic proteasome activity by ethanol may have resulted from adduct formation(s) or oxidative modifications of the proteasome’s subunit proteins with ethanol-derived acetaldehyde and/or reactions with secondary products (ROS, lipid peroxides) derived from ethanol oxidation. At this time, we cannot explain, despite comparable ROS, TBARS, and SAM:SAH ratios in both betaine-treated groups, why proteasome activity was distinctly better preserved when betaine was simultaneously co-administered with ethanol.

The differential regulation by ethanol and/or betaine of the two major ethanol oxidizing enzymes, ADH and CYP2E1 seen here is intriguing. Enhancement of liver ADH activity by acute ethanol administration was not reversed by betaine administered 2 h prior or simultaneously with ethanol. The enhanced ADH activity by acute ethanol administration indicates either an increased catalytic activity of the existing enzyme (i.e., activation) or a rise in its intracellular content. Indeed, we observed a rise in intracellular ADH content by acute ethanol consumption (data not shown).

Regarding cytochrome P450 2E1, a significant rise in hepatic CYP2E1 activity was observed after ethanol alone was administered to mice. This rise in CYP2E1 activity is related to the high blood ethanol levels (50–60 mM) achieved after the ethanol binge in this study. Roberts et al. demonstrated that ethanol, a CYP2E1 substrate, interacts with and protects CYP2E1, from degradation by the proteasome (Roberts et al., 1995). Such protection, in turn, elevates CYP2E1 activity, as seen here. The elevation in CYP2E1 activity by ethanol likely resulted from a 37% decline in proteasome activity in livers of these animals. In mice that received betaine co-administered with ethanol (EB2), proteasome activity was equal to controls (Figure 7C). However, when betaine was administered 2 h prior to ethanol administration, CYP2E1 activity still remained at control levels even though proteasome activity was lower than controls. We speculate that the latter results reflect a “residual effect” of proteasome “activation” by betaine that occurred 2 hours earlier, causing accelerated CYP2E1 degradation restoring CYP2E1 to steady-state (control) levels.

Regarding lysosomal acid lipase and cathepsin L catalytic activities measurements, our data showed significant decline in the activities of both hydrolases in the isolated lysosomal fraction, which were fully restored by simultaneous betaine administration given with ethanol gavages (EB-2). These are interesting results, especially the restoration of lysosomal hydrolases activities with betaine treatments. While the ethanol-induced reduction in lysosomal hydrolase activity with ethanol consumption has been reported before by us (Kharbanda et al., 1995), this is the first study reporting restoration of activity of these hydrolases with betaine treatment. Since no difference was observed in the activities of these hydrolases in the homogenate fractions among all groups of mice, we speculate that the alterations in the post-translational processing and/or impaired trafficking of these hydrolases to the lysosomes with ethanol, as suggested (Kharbanda et al., 1995), are restored by the methylating agent, betaine.

**FIGURE 10:**
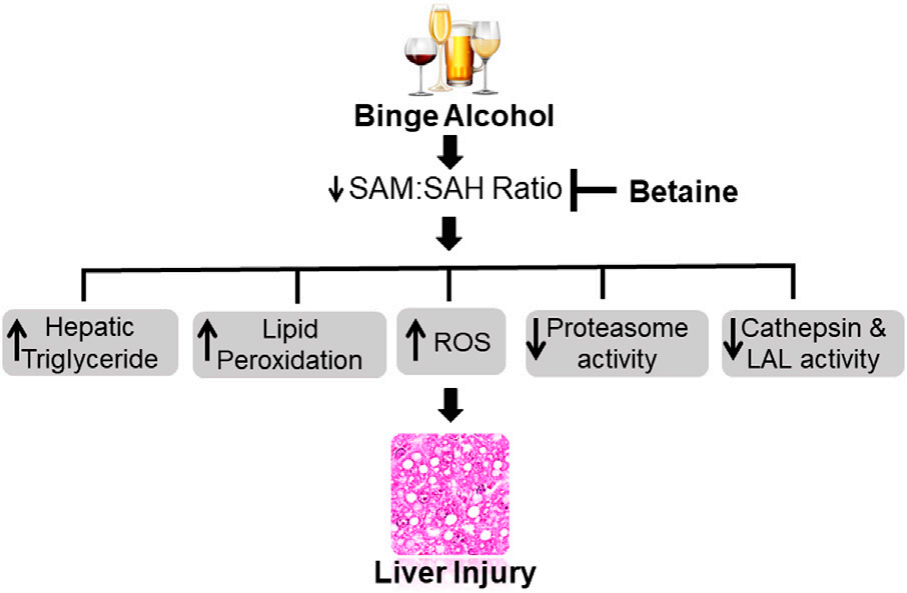
Schematic representation showing acute ethanol-induced liver injury is prevented by betaine administration. Ethanol binges primarily increases hepatocellular S-adenosylhomocysteine (SAH) levels while decreasing S-adenosylmethionine (SAM) generation. The consequent decline in intracellular SAM:SAH ratio causes many detrimental changes in the liver that collectively contribute to the development of liver injury. Betaine co-administration prevents the increase in SAH, promotes SAM generation and by normalizing SAM:SAH ratio attenuates acute ethanol-induced liver damage. ROS - reactive oxygen species, LAL - lysosomal acid lipase.

To summarize, acute ethanol exposure produces similar liver injury, as has been reported before for both acute and chronic ethanol studies. Betaine, whether given 2 h before or simultaneously with ethanol binges significantly attenuated many liver injury parameters measured (liver triglycerides, ROS, TBARS, proteasome activity, serum AST, serum ALT) by regenerating SAM and increasing hepatocellular SAM:SAH ratio as schematically shown in Figure 10. However, simultaneous betaine co-administration showed somewhat better efficacy in preventing liver injury parameters than betaine given 2 h prior to ethanol gavage.

## Conclusion

The present study provided evidence to support the potential therapeutic value of betaine administration in preventing liver injury after ethanol binge drinking.

## Data Availability

All datasets generated for this study are included in the article.

## Abbreviations

AST, aspartate transaminase; ALT, alanine transaminase; BHMT, betaine homocysteine methyltransferase; BAC, blood alcohol concentration; DCFH-DA, 2′7′-dichlorodihydrofluorescein diacetate; DCF, dichlorofluorescein; GNMT, glycine N-methyltransferase; MDA, malondialdehyde; MS, methionine synthase; ROS, reactive oxygen species; SAM, S-adenosylmethionine; SAH, S-adenosylhomocysteine; TBARS, thiobarbituric acid-reactive substances.
